# Development and validation of natural language processing pipelines to extract injury and surgery-related data elements from ACL reconstruction operative reports

**DOI:** 10.1186/s12891-026-10091-w

**Published:** 2026-08-07

**Authors:** Kathleen M. Poploski, Bicen Wang, Scott Rothenberger, Galen Switzer, Sahil Dadoo, Ting Cong, Justin J. Greiner, Jonathan D. Hughes, Volker Musahl, James J. Irrgang, Richard D. Boyce

**Affiliations:** 1 Department of Physical Therapy, University of Pittsburgh, Pittsburgh, PA USA; 2 School of Computing and Information, University of Pittsburgh, Pittsburgh, PA USA; 3 Division of General Internal Medicine, University of Pittsburgh School of Medicine, Pittsburgh, PA USA; 4 Center for Research on Health Care Data Center, University of Pittsburgh School of Medicine, Pittsburgh, PA USA; 5 Department of Orthopaedic Surgery, University of Pittsburgh, Pittsburgh, PA USA; 6 UPMC Freddie Fu Sports Medicine Center, Pittsburgh, PA USA; 7 University of Nebraska Medical Center, Omaha, NE USA; 8 Department of Orthopaedics, Institute of Clinical Sciences, Sahlgrenska Academy, University of Gothenburg, Gothenburg, Sweden; 9 Department of Biomedical Informatics, University of Pittsburgh, Pittsburgh, PA USA

## Abstract

**Background:**

Over 100,000 patients undergo ACLR each year in the United States, providing rich electronic medical record data to improve outcomes, but manual chart review can be time-consuming and expensive. Natural language processing (NLP) methods have been successfully used to extract information for other orthopaedic operative procedures (e.g., hip and knee arthroplasties). Different subspecialities, however, use diverse terminology or “sublanguages” to describe clinical concepts and NLP models perform better concept extraction when trained on specialty-specific texts. Therefore, the purpose of this study was to develop and validate a reliable NLP pipeline to extract meaningful clinical data elements (e.g., graft type, meniscal involvement) from ACLR operative reports.

**Methods:**

Operative reports for a training and test set were randomly selected based on surgeon volume and year from individuals who underwent ACLR within a single healthcare system between 2013 and 2021. Clinical Language Annotation, Modeling, and Processing Toolkit (CLAMP) was utilized to train a domain-specific model and build a pipeline for data extraction of clinically meaningful data elements, including injury-related factors and surgical factors. Relevancy metrics were calculated using values identified by a single clinician as the gold standard.

**Results:**

Overall, the individuals selected for the training and test sets (*n* = 437 total) were 26.6 ± 10.9 years old, and 43.5% were female, similar to the age (26.7 ± 11.5 years) and proportion of females (43.0%) in the full data set (*n* = 5,818). Priority entities, including the side of surgery, ACL graft type, ACL procedure, and meniscal involvement, were identified with F1 scores between 0.87-1.

**Conclusions:**

F1 scores of 0.87-1 were attained for several priority entities using a modest set of annotated ACL operative reports to train the model and an NLP tool designed for non-expert use. The NLP pipelines have the potential to extract relevant operative information for a large cohort of patients to support clinical research, such as identifying predictors of subsequent surgery after ACLR. This work can also inform the planning of future studies using larger training sets and more advanced NLP methods.

**Supplementary Information:**

The online version contains supplementary material available at 10.1186/s12891-026-10091-w.

## Introduction

Over 100,000 patients undergo anterior ligament reconstruction (ACLR) each year in the United States of America. While surgical techniques and rehabilitation have evolved and improved over the last 2 decades, 1 in 5 patients will undergo a subsequent knee surgery within 6 years of ACLR [1, 2]. Risk factors for unplanned subsequent knee surgeries following ACLR are complex and multifactorial. Modifiable and clinically actionable factors, including surgical factors, are of particular interest given the potential to guide intervention. While diagnosis and procedure codes capture some surgical information and have been utilized in prior research studies, they often lack accuracy and detail (e.g., graft type, meniscal repair technique) [3]. Much of the surgical information of interest can only be found in operative reports captured via dictated free text. To utilize surgical details for research purposes, information must typically be prospectively collected via study-specific forms or manually extracted via chart review, both of which can entail significant time and costs.

Natural language processing (NLP), a subset of artificial intelligence (AI) aimed at understanding and interpreting, human language [4], offers promising methods for information extraction from electronic medical records, including automating extraction of granular surgical details at scale. In orthopaedic research, NLP has been used to extract operative information for patients undergoing hip and knee arthroplasties, with studies reporting > 90% accuracy in extracting relevant entities [5–7]. Different subspecialities, however, use diverse terminology or “sublanguages” to describe clinical concepts and NLP models perform better concept extraction when trained on specialty-specific texts than on broad medical texts alone [8]. Given limited research on the application of NLP to operative reports related to ACLR, the purpose of this study was to develop and validate a reliable NLP pipeline to extract meaningful clinical data elements from ACL operative reports.

## Materials and methods

### Dataset overview

**Fig. 1:**
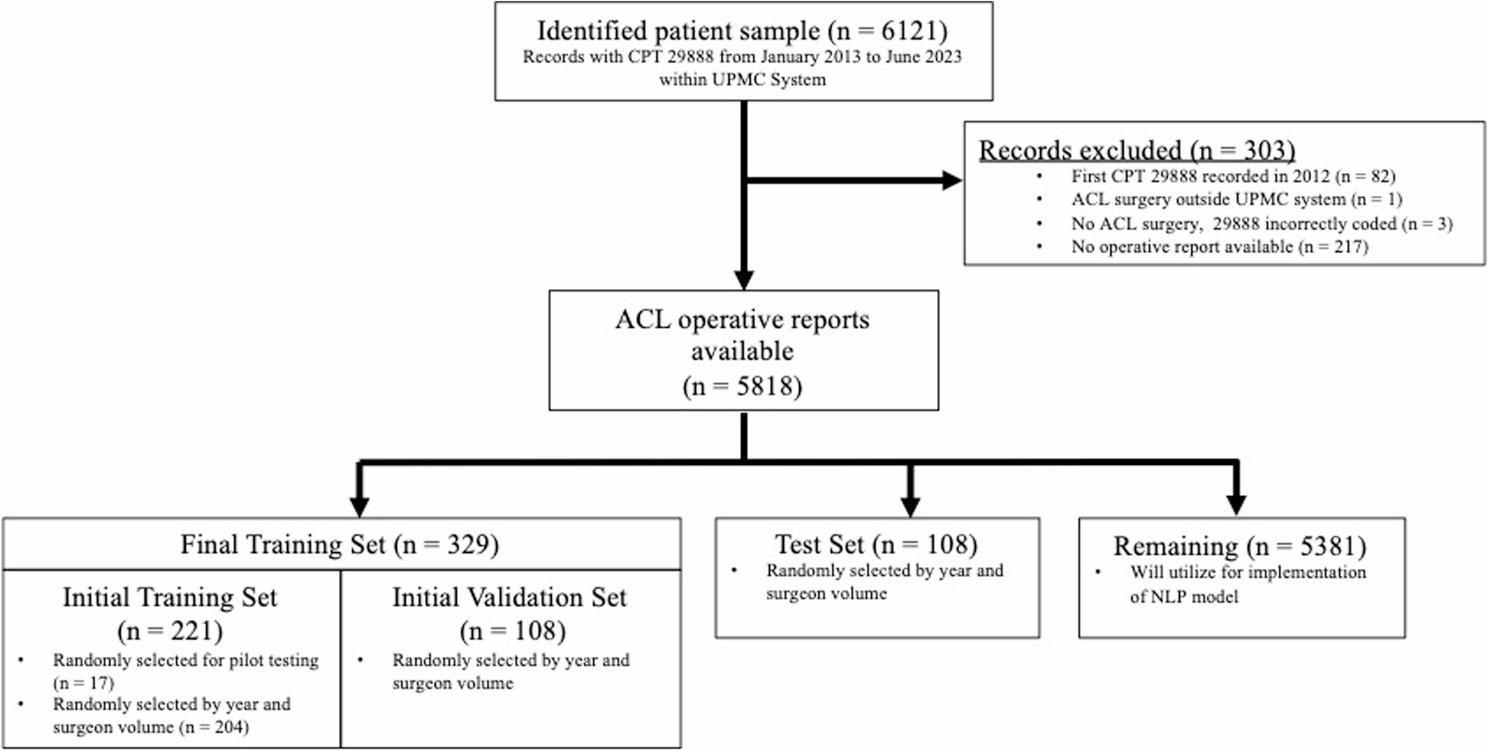
Flow Chart for Operative Report Selection. ACL, anterior cruciate ligament; CPT, Current Procedural Terminology; NLP, natural language processing

This retrospective cross-sectional, methods development and validation study was reviewed by the Institutional Review Board at the University of Pittsburgh (STUDY21030078) and determined to meet the regulatory requirements for exempt research under 45 CFR 46.104. Per the aforementioned institutional review board guidelines and review, informed consent to participate was not required from study participants. Data was obtained from the Health Record Research Request (R3) service of the Department of Biomedical Informatics [9]. Structured fields and unstructured clinical notes from the medical record were extracted for individuals (≥ 14 years old) with at least one record of Current Procedural Code (CPT) 29,888 (“Arthroscopically aided anterior cruciate ligament repair/augmentation or reconstruction”) within the UPMC Health Care System between January 2013 and June 2021. Patients with a record of CPT 29,888 prior to 2013 at a UPMC facility were excluded. After excluding 303 cases that were erroneously included (e.g., miscoded shoulder procedure) or missing operative reports (Fig. 1), ACL operative reports from 5,815 individuals were available for this study.

To ensure the privacy and safety of the dataset, all data were stored on a secure server approved to host clinical research data. The data were de-identified and a study identification number linking the de-identified data with identifiers was maintained separately and securely from the de-identified data. Prior to providing the unstructured clinical notes, R3 used a text combing tool to remove identifiers.

### NLP pipeline development and evaluation

**Fig. 2:**
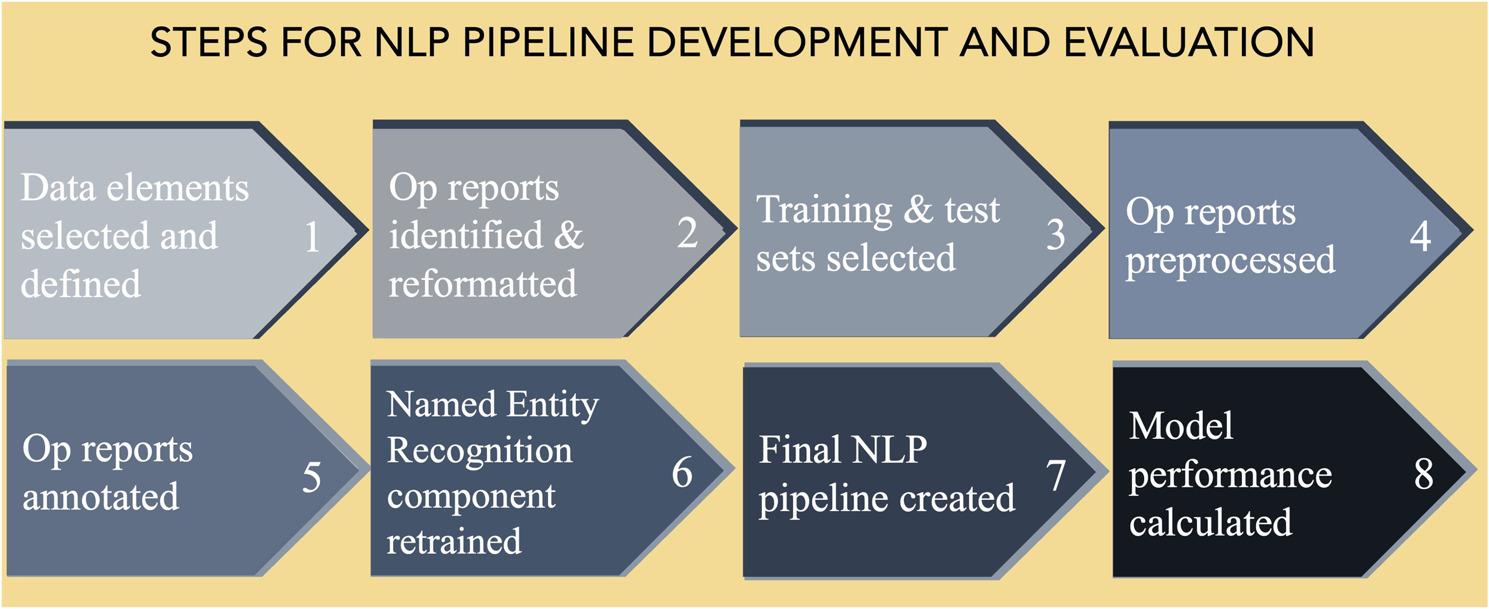
Steps for NLP Pipeline Development and Evaluation. NLP, natural language processing

This study used two NLP tools: CLAMP, Clinical Language Annotation, Modeling and Processing Tool Kit (CLAMP Linux GUI version 1.6.4 and CLAMP Command Line version 1.6.6) [10], and LANN (LANN Enterprise version). CLAMP is a clinical NLP software with components that can be customized to build NLP pipelines for individual use cases. It is specifically designed for clinical note processing and non-NLP expert use, as components can be added to the pipeline and customized via a graphical user interface (GUI). A named entity recognition (NER) component can be customized to identify specific concepts by re-training on annotated or manually labeled texts. LANN software was used to annotate the operative reports. A brief overview of each step is provided in Fig. 2 and below, while details are provided in Appendix 1.

#### Selection of ACL operative report data elements

A list of data elements for extraction was developed and refined through an iterative process based on literature review, expert input including orthopaedic surgeons and researchers, and pilot work with a focus on potential predictors of subsequent surgery following ACLR. Data elements of interest included temporal information (e.g., date of birth and date of surgery) to confirm that the correct operative report was identified and surgical information (See Appendix 2 for full list). Priority entities for extraction included side of surgery, ACL procedure (ACL reconstruction, revision, etc.), ACL graft type, and meniscal involvement. Each data element was entered as an “entity” or label in the annotation schema/framework in LANN. Entities were grouped into categories as appropriate (e.g., ACL diagnosis could be classified as tear, recurrent tear, or avulsion fracture).

#### Operative report identification

The first occurrence of CPT 29888 and the final version of the associated operative report were identified for each individual.

#### Selection of operative reports for training, validation, and test sets

Subsets of ACL operative reports were randomly selected to re-train the NER component (training set), refine performance (validation set), and test performance (test set). Based on pilot work and the literature, it was determined that at least 200 notes were needed in the training set. To ensure variability in procedures, documentation, and changes over time, operative reports were first stratified by surgeon volume and year of surgery. Each note was categorized as being from a high (> 150 total operative reports in the dataset), medium (75–150 total operative reports), or low (< 75 total operative reports) volume surgeon. For the initial training set, eight operative reports per surgeon volume classification per year were randomly selected, except for 2021, where four notes were selected per volume classification, as there was only a half year of records. SAS (SAS Institute Inc. Version 9.4) was used for random note selection. This resulted in 204 total notes for annotation in the initial training set (8 notes * 3 volume classifications * 8.5 years = 204 total notes). These 204 notes plus 17 randomly selected notes for piloting were combined for a total of 221 notes in the initial training set (Fig. 1). Using a similar logic, operative reports were randomly selected for an initial validation set (*n* = 108) and test set (*n* = 108).

#### Operative report preprocessing

CLAMP includes several built-in pipelines for preprocessing clinical text. We started with the General Clinical Concept Extraction pipeline (Clamp-NER), which includes components for sentence detection, tokenization, section identification, parts of speech tagging, dictionary look-up, and assertion classification. We then customized several components of a general concept extraction pipeline based on pilot work. For the sentence detection and section identifier components, we added ACL operative report-specific section headers. Likewise, the dictionary look-up component was modified to include expected phrases and the associated entity label. All notes in the training, validation, and test sets were processed using this pre-annotation pipeline to expedite annotation and facilitate retraining of the NER component.

#### Operative report annotation

**Fig. 3:**
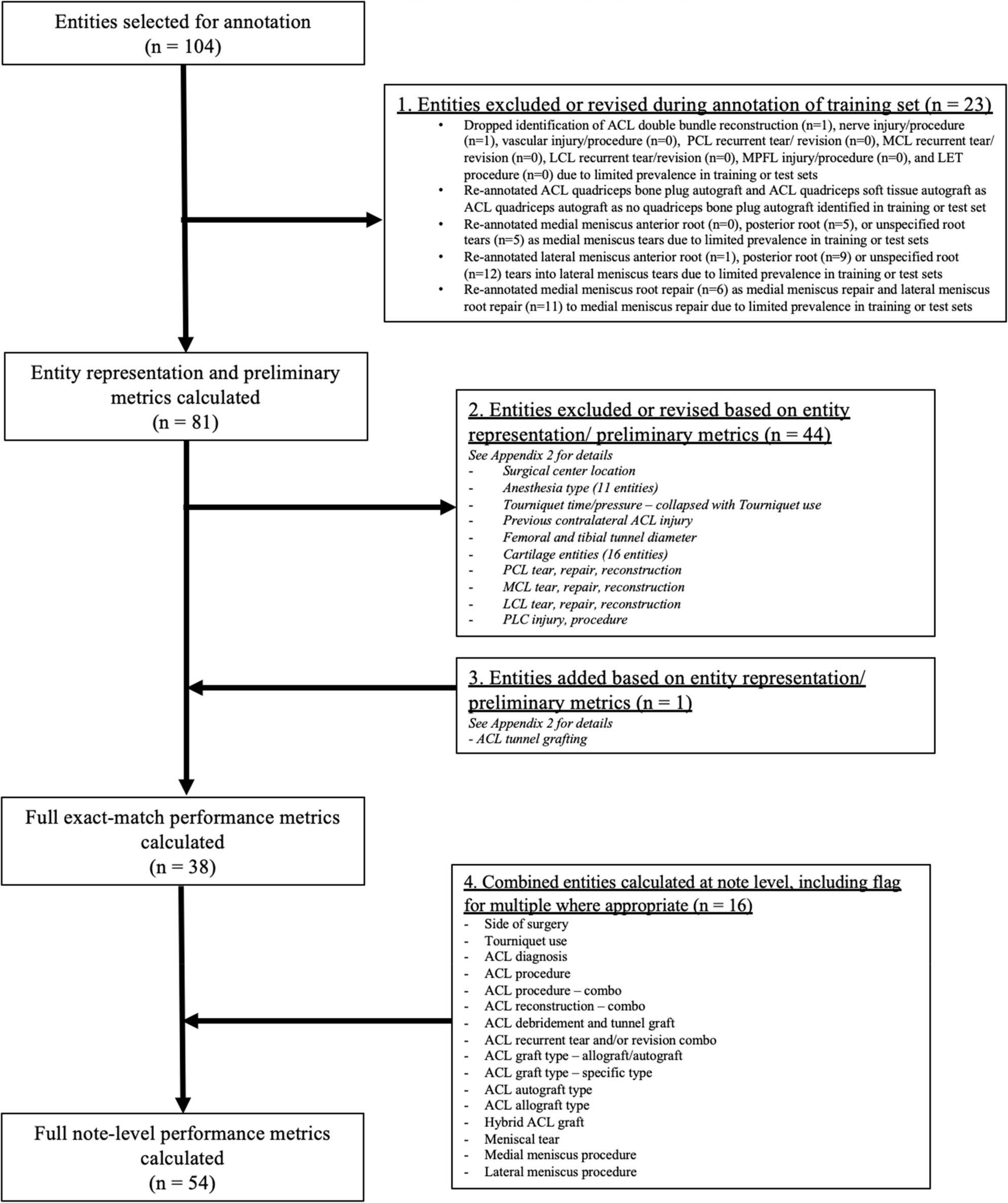
Flowchart for Entity Refinement. ACL, anterior cruciate ligament; ARIF, arthroscopic reduction and internal fixation; LCL, lateral collateral ligament; LET, lateral extra-articular tenodesis; MCL, medial collateral ligament; MPFL, medial patellar femoral ligament; PCL, posterior cruciate ligament; PLC, posterolateral corner

A single clinician-researcher (licensed physical therapist and PhD student with focus on ACL and multiple-ligament knee injuries) annotated all selected notes in LANN, and any questions were discussed and resolved with an orthopaedic surgeon. Following the annotation of the initial training set, several categories of entities were dropped or specific entities reclassified due to limited frequency to simplify the model (Fig. 3 – Box 1).

#### Retraining of NER component

The Conditional Random Fields (CRF) based NER component of the pipeline was retrained using the 221 annotated operative reports from the initial training set and CLAMP’s machine-learning model-building capability.

#### Final NLP pipeline development

A pipeline with similar components to those used for pre-annotation in Step 4 was built with the retrained CRF-based NER component from Step 6 added.

#### Model performance

Pipeline performance was assessed using recall (sensitivity), precision (positive predictive value), and balanced F1 score (harmonic mean of recall and precision). Initial performance metrics were calculated using 108 notes from the initial validation set. Additional entities were eliminated (Fig. 3 – Box 2), and we developed and refined rule-based logic for combined note-level entities (Fig. 3 – Box 4). The initial training and initial validation sets were then combined to create the final training set, which was used to retrain the final NER components (repeated Steps 6–8). Final performance metrics were evaluated using (1) 5-fold cross-validation of the final training set and (2) the test set. Analysis was completed using CLAMP, PostgreSQL, and R Studio (2022.12.0 Build 353).

Exact-match performance and note-level performance metrics were calculated as follows:

- *For Exact match performance*, the word or phrase identified during manual annotation (“gold standard” or ground truth for comparison) had to exactly match the model prediction to be considered correct. An exact match is necessary for some variables, such as the date of surgery. Exact match performance metrics were calculated for the initial validation set and test set. The mean and confidence interval for the performance metrics from 5-fold cross-validation (built-in feature of CLAMP) of the final training set were also calculated to evaluate the performance variability. Confidence intervals were calculated assuming a normal distribution and truncated at 0 if the lower confidence interval was less than 0 or 1 if the upper confidence interval was greater than 1.
- *Note-level performance* was calculated for the combined note-level entities to evaluate the ability of the model to correctly classify a procedure within specified categories based on the entire context of the operative report. Note-level performance metrics were relevant for data elements such as graft type, as this information is often mentioned many times throughout the note, and different phrases can be used to determine classification. Further, graft types that were considered but not used may be mentioned. Note-level performance describes the ability of the model to identify the final overall graft type utilized in the procedure compared to manual identification.

### Source of funding

Funding for this study was provided by the National Center for Advancing Translational Sciences of the NIH, Grant/Award Number: TL1TR001858 (KMP) and Department of Defense (DOD) STaR Trial (W81XWH-17-2-07). Opinions, interpretations, conclusions, and recommendations are those of the author and are not necessarily endorsed by the NIH or the DOD.

## Results

Patient and clinical characteristics of the initial training, initial validation, test sets, and full data set are presented in Table 1. Overall, the individuals selected for this analysis (*n* = 437) were 26.6 ± 10.9 years old, and 43.5% were female, similar to the age (26.7 ± 11.5 years) and proportion of females (43.0%) in the full data set (*n* = 5,818). There was some variability across characteristics and analysis sets, such as females making up 39.5% of the final training set vs. 55.6% of the final test set.

**Table 1:** Patient and Clinical Characteristics of Training, Validation, and Test Sets

	Training Set						Training Set (*n* = 108)		Overall (*n* = 437)		Full Data Set (*n* = 5818)	
	Initial Training Set (*n* = 221)		Initial Validation Set (*n* = 108)		Final Training Set^3^ (*n* = 329)							
	*n*		*n*		*n*		*n*		*n*		*n*	
Age, mean ± sd (y)	221	26.6 ± 11.1	108	27.9 ± 11.4	329	27.0 ± 11.2	108	25.5 ± 10.1	437	26.6 ± 10.9	5818	26.7 ± 11.5
Female	96	43.4%	34	31.5%	130	39.5%	60	55.6%	190	43.5%	2503	43.0%
Surgeons represented^1^	59	66.3%	49	55.0%	66	74.2%	47	52.8%	68	76.4%	89	100%
Year of surgery												
2013	25	11.3%	12	11.1%	37	11.2%	12	11.1%	49	11.2%	755	13.0%
2014	26	11.8%	12	11.1%	38	11.6%	12	11.1%	50	11.4%	743	12.8%
2015	25	11.3%	12	11.1%	37	11.2%	12	11.1%	49	11.2%	673	11.6%
2016	29	13.1%	12	11.1%	41	12.5%	12	11.1%	53	12.1%	737	12.7%
2017	26	11.8%	12	11.1%	38	11.6%	12	11.1%	50	11.4%	680	11.7%
2018	25	11.3%	12	11.1%	37	11.2%	12	11.1%	49	11.2%	684	11.8%
2019	27	12.2%	12	11.1%	39	11.9%	12	11.1%	51	11.7%	706	12.1%
2020	26	11.8%	12	11.1%	38	11.6%	12	11.1%	50	11.4%	513	8.8%
2021	12	5.4%	12	11.1%	24	7.3%	12	11.1%	36	8.2%	327	5.6%
Surgeon volume												
High	80	36.2%	37	34.3%	117	35.6%	36	33.3%	153	35.0%	3357	57.7%
Medium	73	33.0%	35	32.4%	108	32.8%	36	33.3%	140	32.0%	1399	24.0%
Low	68	30.8%	36	33.3%	104	31.6%	36	33.3%	144	33.0%	1062	18.3%
Concomitant meniscus^2^	111	49.8%	62	57.4%	173	52.6%	64	59.3%	237	54.2%	3081	53.0%
Concomitant cartilage^2^	9	4.1%	7	6.5%	16	4.9%	4	3.7%	20	4.6%	256	4.4%

*CPT* Current Procedural Terminology, *sd* standard deviation

^1^ Percents are reported out of 89 total surgeons represented in total dataset

^2^ Identified by relevant CPT codes

^3^ Final training set is composed of 221 notes from the initial training set and 108 notes from the initial validation set for a combined total of 329 notes

### Entity refinement

Initially, 104 entities were selected and defined for potential extraction from ACL operative reports. Through annotation of the initial training set, 23 entities were excluded or revised due to limited presentation or challenges with annotation (Fig. 3). An additional 44 entities were dropped, and one entity was added after the annotation of the initial training and validation sets. Entities were dropped based on several factors, including (1) limited frequency in the initial training/validation sets (Table 2), (2) poor preliminary performance metrics (Appendix 3) and (3) alternative sources of information/importance to the final use case. Some of these entities (e.g., posterior cruciate ligament reconstruction) would have been used to exclude individuals with multiple ligament knee injuries from the dataset for our final use case. As there are alternative methods to identify this information (i.e., procedure codes), we excluded these entities and focused on other entities that cannot be extracted from structured data within the database. Specific reasons for excluding entities are outlined in Appendix 2. Entities with low prevalence but that were part of a category essential to the use case were retained. For example, despite limited representation in the final training set, we retained and reported on entities such as ACL repair, arthroscopic reduction and internal fixation (ARIF), and augmentation as they are part of the overall ACL procedure category. An additional 16 entities, rule-based entities determined from combinations of individual entities, were calculated at the note level. Therefore, final performance metrics were calculated for 38 exact-match entities and 54 note-level entities.

**Table 2:** Frequency of Entities in Training and Test Sets (Note Level)

	Training Set			Test Set (*n* = 108)	Overall (*n* = 437)
	Initial Training Set (*n* = 221)	Initial Validation Set (*n* = 108)	Final Training Set^1^ (*n* = 329)		
Presence of date of birth	221 (100%)	108 (100%)	329 (100%)	108 (100%)	437 (100%)
Presence of date of surgery	220 (99.5%)	107 (99.1%)	327 (99.4%)	106 (98.1%)	433 (99.1%)
*Side of surgery*	220 (99.5%)	108 (100%)	328 (99.7%)	108 (100%)	436 (99.8%)
Right knee	91 (41.2%)	58 (53.7%)	149 (45.3%)	52 (48.1%)	201 (46.0%)
Left knee	129 (58.4%)	50 (46.3%)	179 (54.4%)	56 (51.9%)	235 (53.8%)
*Tourniquet (confirmed used/not used)*	167 (75.6%)	81 (75.0%)	248 (75.4%)	81 (75.0%)	329 (75.3%)
Tourniquet used	131 (59.3%)	67 (62.0%)	198 (60.2%)	67 (62.0%)	265 (60.6%)
Tourniquet not used	36 (16.3%)	14 (13.0%)	50 (15.2%)	14 (13.0%)	64 (14.6%)
*ACL diagnosis*	220 (99.5%)	107 (99.1%)	327 (99.4%)	108 (100%)	435 (99.5%)
Tear	198 (89.6%)	97 (89.8%)	295 (89.7%)	96 (88.9%)	391 (89.5%)
Recurrent tear	20 (9.0%)	10 (9.3%)	30 (9.1%)	12 (11.1%)	42 (9.6%)
Avulsion fracture	2 (0.9%)	0 (0.0%)	2 (0.6%)	0 (0.0%)	2 (0.5%)
*ACL procedure*	219 (99.1%)	107 (99.1%)	326 (99.1%)	108 (100%)	434 (99.3%)
Reconstruction	196 (88.7%)	97 (89.8%)	293 (89.1%)	93 (86.1%)	386 (88.3%)
Repair	1 (0.5%)	0 (0.0%)	1 (0.3%)	0 (0.0%)	1 (0.2%)
Debridement of ACL	0 (0.0%)	0 (0.0%)	0 (0.0%)	2 (1.9%)	2 (0.5%)
ARIF	1 (0.5%)	0 (0.0%)	1 (0.3%)	0 (0.0%)	1 (0.2%)
Augmentation	1 (0.5%)	0 (0.0%)	1 (0.3%)	1 (0.9%)	2 (0.5%)
Revision ACLR	16 (7.2%)	7 (6.5%)	23 (7.0%)	10 (9.3%)	33 (7.6%)
* Graft debridement and tunnel graft*	4 (1.8%)	3 (2.8%)	7 (2.1%)	2 (1.9%)	9 (2.1%)
Graft debridement	4 (1.8%)	3 (2.8%)	7 (2.1%)	2 (1.9%)	9 (2.1%)
ACL tunnel graft	2 (0.9%)	3 (2.8%)	5 (1.5%)	2 (1.9%)	7 (1.6%)
Notchplasty	66 (29.9%)	37 (34.3%)	103 (31.3%)	28 (25.9%)	131 (30.0%)
*ACL graft type*	220 (99.5%)	108 (100%)	328 (99.7%)	108 (100%)	436 (99.8%)
* ACL autograft*	140 (63.3%)	69 (63.9%)	209 (63.5%)	69 (63.9%)	278 (63.6%)
BTB autograft	74 (33.5%)	32 (29.6%)	106 (32.2%)	39 (36.1%)	145 (33.2%)
Hamstring tendon autograft	43 (19.5%)	22 (20.4%)	65 (19.8%)	18 (16.7%)	83 (19.0%)
Quadriceps tendon autograft	23 (10.4%)	15 (13.9%)	38 (11.6%)	12 (11.1%)	50 (11.4%)
* ACL allograft*	78 (35.3%)	38 (35.2%)	116 (35.3%)	36 (33.3%)	152 (34.8%)
Achilles tendon allograft	4 (1.8%)	5 (4.6%)	9 (2.7%)	6 (5.6%)	15 (3.4%)
BTB allograft	11 (5.0%)	8 (7.4%)	19 (5.8%)	6 (5.6%)	25 (5.7%)
Hamstring tendon allograft	14 (6.3%)	6 (5.6%)	20 (6.1%)	7 (6.5%)	27 (6.2%)
Peroneus longus tendon allograft	0 (0%)	2 (1.9%)	2 (0.6%)	0 (0.0%)	2 (0.5%)
Anterior tibial tendon allograft	19 (8.6%)	4 (3.7%)	23 (7.0%)	5 (4.6%)	28 (6.4%)
Posterior tibial tendon allograft	16 (7.2%)	4 (3.7%)	20 (6.1%)	5 (4.6%)	25 (5.7%)
Allograft, NOS	14 (6.3%)	9 (8.3%)	23 (7.0%)	7 (6.5%)	30 (6.9%)
* ACL hybrid graft –* auto/allograft included in respective counts	4 (1.8%)	2 (1.9%)	6 (1.8%)	1 (0.9%)	7 (1.6%)
* No graft – ARIF*,* repair*, *debridement of ACL*,* or ACL graft*^*2*^	6 (2.7%)	3 (2.8%)	9 (2.7%)	4 (3.7%)	13 (3.0%)
Presence of ACL graft width	159 (71.9%)	76 (70.4%)	235 (71.4%)	83 (76.9%)	318 (72.8%)
*Meniscal tear (medial and/or lateral)*	133 (60.2%)	71 (65.7%)	204 (62.0%)	76 (70.4%)	280 (64.1%)
* Medial meniscus tear only* ^*3*^	46 (20.8%)	24 (22.2%)	70 (21.3%)	26 (24.1%)	96 (22.0%)
* Lateral meniscus tear only* ^*3*^	47 (21.3%)	22 (20.4%)	69 (21.0%)	27 (25.0%)	96 (22.0%)
* Medial & lateral meniscus tears* ^*3*^	40 (18.1%)	25 (23.1%)	65 (19.8%)	23 (23.1%)	88 (20.1%)
Medial meniscus tear (all)	86 (38.9%)	49 (45.4%)	135 (41.0%)	49 (45.4%)	184 (42.1%)
Lateral meniscus tear (all)	87 (39.4%)	47 (43.5%)	134 (40.7%)	50 (46.3%)	184 (42.1%)
*Medial meniscus procedure*	71 (32.1%)	48 (44.4%)	119 (36.2%)	42 (38.9%)	161 (36.8%)
Meniscectomy	34 (15.4%)	30 (27.8%)	64 (19.5%)	17 (15.7%)	81 (18.5%)
Repair	40 (18.1%)	19 (17.6%)	59 (17.9%)	25 (23.1%)	84 (19.2%)
Transplant	0 (0%)	0 (0%)	0 (0%)	1 (0.9%)	1 (0.2%)
*Lateral meniscus procedure*	72 (32.6%)	41 (38.0%)	113 (34.3%)	40 (37.0%)	153 (35.0%)
Meniscectomy	47 (21.3%)	29 (26.9%)	76 (23.1%)	29 (26.9%)	105 (24.0%)
Repair	28 (12.7%)	12 (11.1%)	40 (12.2%)	12 (11.1%)	52 (11.9%)
Transplant	0 (0%)	0 (0%)	0 (0%)	0 (0%)	0 (0%)
Synovectomy	13 (5.9%)	4 (3.7%)	17 (5.2%)	10 (9.3%)	27 (6.2%)

Italicized rows are not individual entities, rather combinations or collapsing of individual entities listed in adjacent section at the note level

*ACL* anterior cruciate ligament, *ARIF* arthroscopic reduction and internal fixation, *BTB* bone patellar tendon bone, *NOS* not otherwise specified

^1^ Final training set is composed of 221 notes from the initial training set and 108 notes from the initial validation set for a combined total of 329 notes

^2^ Included for completeness but not an entity; no performance metrics calculated

^3^ Not individual entities – individual performance metrics not calculated

### NLP pipeline performance

The frequencies of the final entities selected for extraction are presented in Table 2, and the final performance metrics are presented in Table 3. Exact-match performance was typically lower than note-level match across all entities. Test set exact-match performance metrics were comparable to cross-validation exact-match metrics.

**Table 3:** Final Performance Metrics

	Final Training Set^1^ – Cross Validation (*n* = 329)				Test Set (*n* = 108)							
	Exact Match Mean (CI)				Exact Match				Note Level Match			
	*n*	Recall	Precision	F1	*n*	Recall	Precision	F1	*n*	Recall	Precision	F1
Date of birth	329	1	1	1	108	1	1	1	108	1	1	1
Date of surgery	328	0.994 (0.983, 1)	1	0.997 (0.991, 1)	106	1	1	1	106	0.991	1.000	0.995
*Side of surgery*									108	1	1	1
Right knee	566	0.811 (0.760, 0.863)	0.845 (0.815, 0.876)	0.828 (0.799, 0.856)	255	0.761	0.951	0.845	52	1	0.929	0.963 0.982
Left knee	715	0.869 (0.824, 0.915)	0.889 (0.827, 0.951)	0.878 (0.845, 0.911)	253	0.692	0.822	0.751	56	1	0.966	
*Tourniquet use*									81	0.938	0.962	0.950
Tourniquet used	236	0.523 (0.431, 0.614)	0.573 (0.468, 0.678)	0.546 (0.452, 0.641)	83	0.458	0.500	0.478	67	0.985	0.957	0.971 0.923
Tourniquet not used	56	0.731 (0.549, 0.914)	0.922 (0.760, 1)*	0.811 (0.650, 0.972)	16	0.500	0.615	0.552	14	0.857	1	
*ACL diagnosis*									108	0.963	0.963	0.963
Tear	1015	0.628 (0.590, 0.666)	0.646 (0.618, 0.673)	0.636 (0.607, 0.666)	345	0.681	0.677	0.679	96	1	0.941	0.970
Recurrent tear	79	0.299 (0.183, 0.416)	0.714 (0.434, 0.994)	0.419 (0.264, 0.573)	38	0.316	0.857	0.462	12	0.583	1	0.737
Avulsion fracture	6	0	NA	0	0	-	-	-	0	-	-	-
*ACL procedure* ^*2*^ *ACL procedure – combo* ^*3*^									108 108	0.889 0.935	0.923 0.971	0.906 0.953
Reconstruction	548	0.898 (0.865, 0.931)	0.864 (0.837, 0.892)	0.881 (0.858, 0.903)	174	0.891	0.829	0.859	93	0.978	0.883	0.929
* Reconstruction – combo*^*3*^									93	0.979	0.961	0.973
Repair	1	0	NA	0	0	-	-	-	0	-	-	-
Debridement of ACL	0	-	-	-	2	0	NA	0	2	0	NA	0
ARIF	1	0	NA	0	0	-	-	-	0	-	-	-
Augmentation	1	0	NA	0	2	0	NA	0	1	0	NA	0
Revision ACLR	34	0.565 (0.514, 0.617)	0.609 (0.486, 0.732)	0.582 (0.521, 0.643)	14	0.286	1	0.444	10	0.500	1	0.667
* Recurrent tear/revision combo*^*3*^									12	0.833	1	0.909
* Graft debridement and tunnel graft*									2	0	NA	0
Graft debridement	9	0	NA	0	2	0	NA	0	2	0	NA	0
ACL tunnel grafting	12	0	NA	0	5	0	NA	0	2	0	NA	0
Notchplasty	117	0.728 (0.584, 0.871)	0.742 (0.634, 0.849)	0.734 (0.609, 0.859)	32	0.906	0.853	0.879	28	1	1	1
*ACL graft type – allograft/autograft* ^*4*^									104	0.913	0.950	0.931
*ACL graft type – specific* ^*5*^									104	0.885	0.911	0.898
* ACL autograft*									69	0.913	0.926	0.920
BTB autograft	202	0.721 (0.656, 0.786)	0.765 (0.704, 0.827)	0.740 (0.704, 0.777)	81	0.691	0.862	0.767	39	0.974	0.884	0.927
Hamstring tendon autograft	160	0.592 (0.471, 0.713)	0.720 (0.590, 0.850)	0.647 (0.531, 0.764)	44	0.659	0.906	0.763	18	0.889	1	0.941
Quadriceps tendon autograft	92	0.798 (0.692, 0.904)	0.906 (0.790, 1)*	0.845 (0.769, 0.920)	29	0.586	0.944	0.723	12	1	1	1
* ACL allograft*									36	0.889	0.889	0.889
Achilles tendon allograft	15	0.558 (0,1)*	0.625 (0.172, 1)*	0.583 (0.055, 1)*	10	0.8	1	0.889	6	1	1	1
BTB allograft	26	0.642 (0.200, 1)*	0.960 (0.849, 1)*	0.712 (0.395, 1)*	9	0.778	1	0.875	6	1	1	1
Hamstring tendon allograft	37	0.605 (0.371, 0.839)	0.950 (0.811, 1)*	0.727 (0.536, 0.918)	12	0.75	0.9	0.818	7	1	0.875	0.933
Peroneus longus tendon allograft	2	0	NA	0	0	-	-	-	0	-	-	-
Anterior tibial tendon allograft	38	0.560 (0.215, 0.906)	0.721 (0.538, 0.904)	0.615 (0.339, 0.891)	6	0.833	1	0.909	5	0.800	1	0.889
Posterior tibial tendon allograft	36	0.607 (0.238, 0.975)	0.822 (0.613, 1)*	0.683 (0.364, 1)	9	0.667	0.857	0.75	5	1	1	1
Allograft, NOS	95	0.569 (0.330, 0.808)	0.684 (0.497, 0.870)	0.616 (0.397, 0.835)	30	0.5	0.6	0.545	7	0.714	0.625	0.667
* ACL hybrid graft* ^*6*^									1	0	0	0
ACL graft width	330	0.541 (0.418, 0.665)	0.694 (0.620, 0.768)	0.605 (0.505, 0.705)	108	0.509	0.679	0.582	83	0.602	0.758	0.671
*Meniscal tear* ^*7*^									76	0.921	0.959	0.940
Medial meniscus tear	407	0.687 (0.634, 0.740)	0.846 (0.797, 0.894)	0.758 (0.711, 0.805)	145	0.759	0.866	0.809	49	0.918	0.978	0.947
Lateral meniscus tear	386	0.641 (0.564, 0.718)	0.764 (0.675, 0.852)	0.695 (0.627, 0.764)	137	0.73	0.826	0.775	50	0.980	1	0.990
*Medial meniscus procedure*									42	0.905	1	0.950
Meniscectomy	106	0.548 (0.473, 0.623)	0.820 (0.760, 0.880)	0.656 (0.593, 0.719)	30	0.6	0.857	0.706	17	0.941	1	0.970
Repair	118	0.636 (0.520, 0.753)	0.875 (0.811, 0.939)	0.733 (0.654, 0.812)	43	0.837	1	0.911	25	0.880	1	0.936
Transplant	0	-	-	-	1	0	NA	0	1	0	NA	0
*Lateral meniscus procedure*									40	0.775	1	0.873
Meniscectomy	126	0.607 (0.558, 0.655)	0.812 (0.737, 0.886)	0.694 (0.638, 0.750)	46	0.630	0.935	0.753	29	0.724	1	0.840
Repair	69	0.773 (0.554, 0.992)	0.901 (0.786, 1)*	0.825 (0.674, 0.976)	18	0.833	0.938	0.882	12	0.917	1	0.957
Transplant	0	-	-	-	0	-	-	-	0	-	-	-
Synovectomy	31	0.655 (0.247, 1)*	0.747 (0.549, 0.944)	0.668 (0.378, 0.958)	17	0.471	0.889	0.615	10	0.900	1	0.947

Italicized rows are not individual entities, rather combinations or collapsing of individual entities listed in adjacent section at the note level

In cross validation and test set exact match n may be greater than the number of notes as entities often appear more than one time in a note

*ACL* anterior cruciate ligament, *ARIF* arthroscopic reduction and internal fixation, *BTB* bone patellar tendon bone, *NOS* not otherwise specified

* Normal distribution assumed for confidence intervals. Truncated at 0 if lower confidence interval less than 0 or truncated at 1 if upper confidence interval greater than 1

^1^ Final training set is composed of 221 notes from the initial training set and 108 notes from the initial validation set for a combined total of 329 notes

^2^ ACL procedure – categories include ACL reconstruction, Debridement of ACL, Augmentation, ACL graft debridement and/or tunnel graft, and ACL revision; ACL repair and ARIF not present in test set

^3^ ACL procedure – combo: Same as overall ACL procedure entity but instead of a revision ACLR category, created a combined category for individuals with indication of recurrent tears, revision ACLR, debridement of grafts or bone tunnel grafting as these individuals will be excluded from final use case

^4^ ACL graft type – allograft/ autograft: collapsed specific graft types to categorize each note as allograft, autograft, or hybrid (3 categories)

^5^ ACL graft type – specific: categorized each note by specific autograft and/or allograft type (11 categories)

^6^ ACL hybrid graft: Autograft and allograft types included in respective counts

^7^ Meniscal tear – specific location: Three categories including medial meniscus tear, lateral meniscus tear, both medial and lateral meniscal tears

At the note level (note-level entities), an F1 score of at least 0.9 was achieved for 67% of the final entities, and an F1 score of at least 0.95 was attained for 41%. Performance metrics were particularly high for structured data such as date of birth/surgery as well as entities such as notchplasty and several ACL graft types, which achieved scores of 1 for recall, precision, and F1. Seven entities, including ACL augmentation, debridement of the ACL, and ACL tunnel grafting, had F1 measures of 0; however, these entities were found with very low frequency in the training and test sets. The final NLP pipeline attained F1 scores between 0.87 and 1 for extraction of priority entities, including ACL procedure (e.g., primary versus revision), side of surgery, graft type, and meniscal involvement.

## Discussion

This study successfully developed and validated an NLP pipeline that achieved F1 scores of 0.87-1.0 for several priority entities using a modest set of annotated ACL operative reports in the training set and an NLP tool designed for non-expert use. The customized pipelines can be utilized to efficiently extract operative report information for a large cohort of patients who underwent ACLR. It can expedite ongoing data capture as new patients under ACLR to enable future quality improvement and clinical research to improve clinical and surgical outcomes more broadly.

### Side of surgery

The side of surgery was unavailable within structured data within our dataset and therefore, essential for determining if subsequent surgeries were to the ipsilateral or contralateral side relative to primary ACLR. Through iterative refinement of the model, it was determined that utilizing a rule-based method where the side with the most mentions/model predictions was selected as the side of surgery reached perfect performance for the determination of side surgery.

### ACL procedure

Differentiating operative reports for primary versus revision ACLR procedures was necessary as there is no specific code to distinguish these procedures. Performance for the combined entity *ACL procedure* appears good (F1 score = 0.91); however, the performance is heavily weighted by the *ACL reconstruction* entity as it was more frequently found than any other ACL procedure category. The combined entity, *ACL procedure – combo*, reached the highest overall performance (F1 score = 0.953) by utilizing several entities, including recurrent tear, revision, and debridement, to identify cases of graft failure.

We conducted an error analysis to understand the potential for using the pipeline to determine the ACL procedure for the full dataset. There were 4 cases where the pipeline did not return any result for the ACL procedure – recurrent/revision combo entity. These were due to instances of low representation in the training set (ACL augmentation case) or nuanced documentation. When applying the model to the full dataset for entity extraction, cases where no ACL procedure or conflicting information is found can be manually reviewed to identify the correct procedure.

### ACL graft type

Different ACL graft types have been associated with an increased risk of ACLR failure and revision surgery [11, 12] as well as other subsequent surgeries, including surgery for painful hardware [13], meniscal injury [2, 14], articular cartilage injury [2], and loss of motion [2]. Perfect performance was achieved for extracting several individual ACL graft types, including quadriceps autograft, Achilles allograft, bone patellar tendon bone (BTB) allograft, and tibialis posterior allograft; however, the most relevant graft entity, *ACL graft type–specific*, which predicted the overall ACL graft type for the note, reached a slightly lower F1 score of 0.90. For full extraction, cases where no ACL graft type or multiple graft types are identified can be manually reviewed, which would have enabled correction of 11/14 (78.6%) of the ACL graft type errors in the final test set.

### Meniscal involvement

Meniscal pathology and associated intervention at the time of ACLR may impact outcomes and the risk of subsequent knee surgery [2, 13, 15–17]. While diagnosis and procedure codes can be used to identify meniscal involvement, differentiation of medial versus lateral meniscus is not always possible. Nearly perfect precision was attained across all meniscal-related entities. There were several instances of false negatives where no medial or lateral meniscal tear or appropriate meniscal procedure was identified by the pipeline. For application to the full dataset, model predictions and CPT codes for meniscal procedures can be cross-referenced to improve data quality.

### Other entities

Date of birth and date of surgery achieved high performance across all metrics. While this information is found in structured data, can be utilized to confirm that the correct operative report is identified for each individual. Acceptable performance metrics were also achieved for tourniquet use, notchplasty, and synovectomy, which achieved performance of ≥ 0.9 for all metrics.

A broad range of data elements was initially considered for extraction, providing valuable information for future studies. Some entities were excluded due to inconsistent documentation in the operative reports. For example, anesthesia information is typically documented in nursing and anesthesia notes and is not always included in operative reports. The methods developed here can be refined through training on anesthesia notes in future iterations. Other entities, such as the location of articular cartilage injury and associated procedures, were dropped due to low frequency in the training set. Information on the frequency of these entities and initial performance can be utilized to inform training/test set sample sizes and selection of methods, including targeted extraction (e.g., identification of operative reports with articular cartilage injuries/procedures via diagnosis or procedure code) and more advanced NLP methods (e.g., deep learning) in future work. Further, documentation practices can vary significantly across departments and institutions. Future work should also test model performance on operative reports from other systems and refine performance through additional training on these sources.

### NLP and orthopaedic research

To date, most research on NLP and ACL injuries has focused on the diagnosis of ACL tears from imaging [18, 19]. There has been some work within orthopaedics to extract information from arthroplasty clinical notes and operative reports. Shah and colleagues extracted relevant entities from preoperative, operative, and postoperative information from patients who underwent total hip or total knee arthroplasty with greater than 91% accuracy across all variables [5]. Wyles et al. achieved great than 90% accuracy for extracting operative approach, fixation method, and bearing surface category from total hip arthroplasty operative notes [7]. Finally, Sagheb achieved > 98% accuracy when extracting four data elements from knee arthroplasty operative reports [6]. Unfortunately, it is difficult to make comparisons with the current study as only one of the described studies reported an F1 score [6] and accuracy may overinflate performance due to a high number of true negatives. Still, these works offer foundational insights into extracting clinical data elements from orthopaedic operative reports. A more recent study used clinical data from a multi-site tertiary-care regional children’s hospital to evaluate the possibility of using NLP to build registries from unstructured clinical notes [20]. 

It is conceivable for NLP methods to be useful for an array of clinical applications. In the future, these methods may be used to identify clinical risk factors within medical records, which are normally too nuanced for conventional data capture, including minor details in surgical methodologies or differences in physical examination. More advanced learning models have the potential to inform implant or instrumentation selection for surgical intervention. Such findings could reduce surgical complication rates and costs by identifying practice patterns that lead to inferior outcomes or negligible improvement.

### Limitations

This study is not without limitations. We were unable to achieve reliable extraction performance for several entities of interest, such as ACL graft width. More advanced NLP methods, including deep learning, may enable improved extraction of entities, particularly low-frequency entities. We chose to use CLAMP for this project as it is specifically designed for extraction from medical notes and non-NLP expert use. While there still is a learning curve to using the tool, we were able to achieve reliable performance for our priority entities of interest using CLAMP. Further, CLAMP and LANN allow for local data storage, enabling us to maintain necessary security restrictions given the sensitive nature of the dataset. Another potential limitation is that we did not use a standardized ontology for labeling entities. During piloting, we attempted to utilize CLAMP’s Unified Medical Language System (UMLS) Encoder feature to pre-annotate notes. This pipeline component matches clinical concepts to UMLS Concept Unique Identifiers (CUIs). Despite efforts to focus on certain vocabularies and semantic types, we found that for every correct label, there were three labels (range 12–50%) that were incorrect or not of interest.

Other limitations include reliance on a single clinician-researcher for report annotation and a relatively small training set (5.6% of the full dataset), leading to some entities with low frequency and an inability to calculate performance metrics. Finally, a normal distribution was assumed for exact match confidence intervals, which may not be accurate for all entities.

## Conclusion

An acceptable level of performance was attained for extracting several priority entities using a modest set of annotated ACL operative reports and an NLP tool designed for non-expert use. The NLP pipelines can be used as a starting model to extract relevant operative information from a large cohort of patients to support clinical research, such as identifying predictors of subsequent surgery after ACLR [21]. This foundational work can also inform the planning of future studies using more advanced natural language processing methods and larger training sets within our institution or other healthcare systems [7]. 

## Supplementary Information

Supplementary Material 1: Appendix 1 Steps for NLP Pipeline Development and Evaluation. Appendix 2 Frequency of Entities in Training Set (Note Level). Appendix 3 Initial Validation Set Performance Metrics.

## Data Availability

The data that support the findings of this study are not publicly available due to reasons of sensitivity and to ensure patient confidentiality but it may be made available from corresponding author if necessary.
