# Supplementary material for: Development and validation of natural language processing pipelines to extract injury and surgery-related data elements from ACL reconstruction operative reports
Source: BMC Musculoskelet Disord. 2026 Aug 7;27:659. doi: 10.1186/s12891-026-10091-w (PMC13449579; doi:10.1186/s12891-026-10091-w)
Supplement: Supplementary file 1 [file 12891_2026_10091_MOESM1_ESM.docx]

**APPENDIX TABLES**

Appendix 1 Steps for NLP Pipeline Development and Evaluation

| Step | Program | Description |
| --- | --- | --- |
| 1 | LANN | **Selection of ACL operative report data elements for annotation and extraction**   - Entities created in LANN based on data elements of interest |
| 2 | DBeaver Relational Database/ PostgreSQL  Bash shell | **Operative report identification and reformatting**   - Identified final version of operative report note associated with first occurrence of CPT 29888 for each individual in dataset - Concatenated notes represented on multiple rows - Exported as .txt file and re-formatted for ease of annotation |
| 3 | SAS | **Selection of operative reports for training, validation, and test sets**   - Randomly selected operative reports for training and test sets based upon surgeon volume and year of surgery |
| 4 | CLAMP | **Operative report preprocessing**  Pre-annotated notes via CLAMP pipeline:   1. Sentence Detector* - *DF_Clamp_sentence_detector* 2. Tokenizer – *DF_Clamp_tokenzier* 3. Parts of speech tagger – *DF_OpenNLP_POS_tagger* 4. Section header identifier* - *DF_Dictionary_based_section_identifier* 5. Chunker – *DF_OpenNLP_chunker* 6. Named entity recognition (NER)* - *DF_Dictionary_lookup* 7. Assertion classifier – *DF_NegEx-assertion*   *customized to dataset based on ACL operative report section headers and key phrases identified from pilot work  *CLAMP xmi file output directly imported to LANN (Step 5)* |
| 5 | LANN  Terminal   - Python script - Java script | **Operative report annotation**   - Annotated initial training, initial validation, and test sets (json output) - Converted LANN json output files to xmi files for import to CLAMP   - Converted LANN json output to ann/txt files   - Converted ann/txt files to xmi for import to CLAMP |
| 6 | CLAMP | **Retraining of NER component using annotated ACL operative reports**  NER feature extractors utilized:   - *DF_Dictionary_lookup_Feature* - *DF_Discrete_Word_Embedding_Feature* - *DF_Ngram_feature* - *DF_Prefix-suffix_feature* - *DF_Random_indexing_feature* - *DF_Section_feature* - *DF_Sentence_feature* - *DF_Sentence_pattern_feature* - *DF_Word_embedding_feature* - *DF_Word_shape_feature* - *DF_Words_regular_expression_feature* |
| 7 | CLAMP | **Extraction pipeline creation**  Added retrained NER component to final pipelines and ran pipelines to extract entities from ACL operative reports  Initially used Pipeline 1 for all entities. When refining pipelines, determined that utilizing 3 pipelines improved performance:  Pipeline 1**.** Used for identification of all entities except those extracted with pipelines 2 and 3. Components utilized:   1. Sentence Detector* - *DF_Clamp_sentence_detector* 2. Tokenizer – *DF_Clamp_tokenzier* 3. Parts of speech tagger – *DF_OpenNLP_POS_tagger* 4. Section header identifier* - *DF_Dictionary_based_section_identifier* 5. Named entity recognition* - *DF_Dictionary_lookup* 6. Named entity recognition - *DF_CRF_based_named_entity_recognizer*** 7. Assertion classifier – *DF_NegEx-assertion*   Pipeline 2. Used for extraction of date birth and date of surgery; different tokenizer and addition of chunker needed for best extraction  Differences from pipeline 1:  - Utilized different Tokenizer: *DF_OpenNLP_tokenizer*  - Added chunker: *DF_OpenNLP_chunker*  - Named entity recognition - *DF_CRF_based_named_entity_recognizer***  trained on date of birth and date of surgery entities only  Pipeline 3. Used for extraction of side of surgery; Due to overlap between side of surgery and other entities, a separate pipeline for extracting left_knee and right_knee entities needed:  Differences from pipeline 1:  - Named entity recognition-*DF_CRF_based_named_entity_recognizer***  trained on left and right knee entities only    * Customized to dataset based on ACL operative report section headers and key phrases identified from pilot work  ** Retrained NER component based on ACL op report corpus |
| 8 | CLAMP  R Studio  Bash shell  SQL | **Model performance**  Exact-match performance metrics calculated  Cross validation performance metrics calculated for each fold  Cross validation mean and CI metrics calculated  Note-level performance metrics calculated   - Extracted individual operative report output and combined into a single file for ground-truth and model-predicted annotations - Calculated note-level performance metrics |
| ACL, anterior cruciate ligament; CI, confidence intervals; CLAMP, Clinical Language Annotation, Modeling, and Processing Toolkit; CPT, Current Procedural Terminology; NER, named entity recognition; NLP, natural language processing | | |

Appendix 2 Frequency of Entities in Training Set (Note Level)

|  |  | **Training Set** |  | **Notes** | **Dropped** |  |
| --- | --- | --- | --- | --- | --- | --- |
|  | **Initial Training Set** (n = 221) | **Initial Validation Set**  (n = 108) | **Final Training Set^1^**  (n = 329) |  |  |  |
| Presence of date of birth | 221 (100%) | 108 (100%) | 329 (100%) |  |  |  |
| Presence of date of surgery | 220 (99.5%) | 107 (99.1%) | 327 (99.4%) |  |  |  |
| Hospital | 55 (24.9%) | 24 (22.2%) | 79 (24.0%) | Annotated to extract satellite centers. Information often redacted so infeasible to accurately extract | X |  |
| *Side of surgery* | 220 (99.5%) | 108 (100%) | 328 (99.7%) |  |  |  |
| Right knee  Left knee | 91 (41.2%)  129 (58.4%) | 58 (53.7%)  50 (46.3%) | 149 (45.3%)  179 (54.4%) |  |  |  |
| *General anesthesia* | 204 (92.3%) | 102 (94.4%) | 306 (93.0%) | Eliminated due limited documentation of specific general anesthesia type | X  X  X |  |
| Endotracheal  Laryngeal mask  General, NOS | 17 (7.7%)  20 (9.0%)  167 (75.6%) | 10 (9.3%)  7 (6.5%)  85 (78.7%) | 27 (8.2%)  27 (8.2%)  252 (76.6%) |  |  |  |
| *Regional anesthesia* | 77 (34.8%) | 37 (34.3%) | 114 (34.7%) | Eliminated due to inconsistent documentation in operative report– often regional anesthesia is administered prior to the patient entering the operating room so it may not be documented in Operative Report | X  X  X  X  X  X  X  X  X |  |
| Spinal  Epidural  Femoral block  Sciatic block  Adductor canal block  *Adductor/sciatic block*  Femoral/sciatic block  *Femoral/spinal*  Regional, NOS | 0 (0%)  0 (0%)  39 (17.6%)  0 (0%)  6 (2.7%)  1 (0.5%)  5 (2.3%)  0 (0%)  26 (11.8%) | 1 (0.9%)  0 (0.0%)  16 (14.8%)  0 (0.0%)  0 (0.0%)  0 (0%)  2 (1.9%)  1 (0.9%)  17 (15.7%) | 1 (0.3%)  0 (0.0%)  55 (16.7%)  0 (0.0%)  6 (1.8%)  1 (0.3%)  7 (2.1%)  1 (0.3%)  43 (13.1%) |  |  |  |
| Local anesthesia | 5 (2.3%) | 2 (1.9%) | 7 (2.1%) | Limited representation | X |  |
| *Tourniquet (confirmed used/not used)* | 167 (75.6%) | 81 (75.0%) | 248 (75.4%) |  |  |  |
| Tourniquet used  Tourniquet not used | 131 (59.3%)  36 (16.3%) | 67 (62.0%)  14 (13.0%) | 198 (60.2%)  50 (15.2%) |  |  |  |
| Presence of tourniquet time | 70 (31.7%) | 33 (30.6%) | 103 (31.3%) | Combined with tourniquet use due to variation in format of documenting quantity and units | X |  |
| Presence of tourniquet pressure | 83 (37.6%) | 51 (47.2%) | 134 (40.7%) |  | X |  |
| *ACL diagnosis* | 220 (99.5%) | 107 (99.1%) | 327 (99.4%) |  |  |  |
| Tear | 198 (89.6%) | 97 (89.8%) | 295 (89.7%) |  |  |  |
| Recurrent tear | 20 (9.0%) | 10 (9.3%) | 30 (9.1%) |  |  |  |
| Avulsion fracture | 2 (0.9%) | 0 (0.0%) | 2 (0.6%) |  |  |  |
| *ACL procedure* | 219 (99.1%) | 107 (99.1%) | 326 (99.1%) |  |  |  |
| Reconstruction | 196 (88.7%) | 97 (89.8%) | 293 (89.1%) |  |  |  |
| Repair | 1 (0.5%) | 0 (0.0%) | 1 (0.3%) |  |  |  |
| Debridement of ACL | 0 (0.0%) | 0 (0.0%) | 0 (0.0%) |  |  |  |
| ARIF | 1 (0.5%) | 0 (0.0%) | 1 (0.3%) |  |  |  |
| Augmentation | 1 (0.5%) | 0 (0.0%) | 1 (0.3%) |  |  |  |
| Revision ACLR | 16 (7.2%) | 7 (6.5%) | 23 (7.0%) |  |  |  |
| *Graft debridement and tunnel graft* | 4 (1.8%) | 3 (2.8%) | 7 (2.1%) |  |  |  |
| Graft debridement | 4 (1.8%) | 3 (2.8%) | 7 (2.1%) |  |  |  |
| ACL tunnel grafting | 2 (0.9%) | 3 (2.8%) | 5 (1.5%) | Added based on entity representation/ preliminary metrics |  |  |
| ACL prior contralateral injury | 6 (2.7%) | 0 (0%) | 6 (1.8%) | Limited representation | X |  |
| Notchplasty | 66 (29.9%) | 37 (34.3%) | 103 (31.3%) |  |  |  |
| *ACL graft type* | 220 (99.5%) | 108 (100%) | 328 (99.7%) |  |  |  |
| *ACL autograft* | 140 (63.3%) | 69 (63.9%) | 209 (63.5%) |  |  |  |
| BTB autograft | 74 (33.5%) | 32 (29.6%) | 106 (32.2%) |  |  |  |
| Hamstring tendon autograft | 43 (19.5%) | 22 (20.4%) | 65 (19.8%) |  |  |  |
| Quadriceps tendon autograft | 23 (10.4%) | 15 (13.9%) | 38 (11.6%) |  |  |  |
| *ACL allograft* | 78 (35.3%) | 38 (35.2%) | 116 (35.3%) |  |  |  |
| Achilles tendon allograft | 4 (1.8%) | 5 (4.6%) | 9 (2.7%) |  |  |  |
| BTB allograft | 11 (5.0%) | 8 (7.4%) | 19 (5.8%) |  |  |  |
| Hamstring tendon allograft | 14 (6.3%) | 6 (5.6%) | 20 (6.1%) |  |  |  |
| Peroneus longus tendon allograft | 0 (0%) | 2 (1.9%) | 2 (0.6%) |  |  |  |
| Anterior tibial tendon allograft | 19 (8.6%) | 4 (3.7%) | 23 (7.0%) |  |  |  |
| Posterior tibial tendon allograft | 16 (7.2%) | 4 (3.7%) | 20 (6.1%) |  |  |  |
| Allograft, NOS | 14 (6.3%) | 9 (8.3%) | 23 (7.0%) |  |  |  |
| *ACL hybrid graft^1^* | 4 (1.8%) | 2 (1.9%) | 6 (1.8%) |  |  |  |
| *No graft – ARIF, repair,*  *debridement of ACL, or ACL graft^2^* | 6 (2.7%) | 3 (2.8%) | 9 (2.7%) |  |  |  |
| Presence of ACL graft width | 159 (71.9%) | 76 (70.4%) | 235 (71.4%) |  |  |  |
| Presence of femoral tunnel diameter | 166 (75.1%) | 75 (69.4%) | 241 3.3%) | Eliminated due to high variability of documentation, low preliminary performance metrics and low priority for final use-case | X |  |
| Presence of tibial tunnel diameter | 165 (74.7%) | 83 (76.9%) | 248 (75.4%) |  | X |  |
| *Meniscal tear (medial and/or lateral)* | 133 (60.2%) | 71 (65.7%) | 204 (62.0%) |  |  |  |
| *Medial meniscus tear only* | 46 (20.8%) | 24 (22.2%) | 70 (21.3%) |  |  |  |
| *Lateral meniscus tear only* | 47 (21.3%) | 22 (20.4%) | 69 (21.0%) |  |  |  |
| *Medial & lateral meniscus tears* | 40 (18.1%) | 25 (23.1%) | 65 (19.8%) |  |  |  |
| Medial meniscus tear | 86 (38.9%) | 49 (45.4%) | 135 (41.0%) |  |  |  |
| Lateral meniscus tear | 87 (39.4%) | 47 (43.5%) | 134 (40.7%) |  |  |  |
| *Medial meniscus procedure* | 71 (32.1%) | 48 (44.4%) | 119 (36.2%) |  |  |  |
| Meniscectomy | 34 (15.4%) | 30 (27.8%) | 64 (19.5%) |  |  |  |
| Repair | 40 (18.1%) | 19 (17.6%) | 59 (17.9%) |  |  |  |
| Transplant | 0 (0%) | 0 (0%) | 0 (0%) |  |  |  |
| *Lateral meniscus procedure* | 72 (32.6%) | 41 (38.0%) | 113 (34.3%) |  |  |  |
| Meniscectomy | 47 (21.3%) | 29 (26.9%) | 76 (23.1%) |  |  |  |
| Repair | 28 (12.7%) | 12 (11.1%) | 40 (12.2%) |  |  |  |
| Transplant | 0 (0%) | 0 (0%) | 0 (0%) |  |  |  |
| *Articular cartilage injury* | 88 (39.8%) | 43 (39.8%) | 131 (39.8%) |  |  |  |
| Medial femoral condyle  Lateral femoral condyle  Trochlea  Medial tibial plateau  Lateral tibial plateau  Patella  Patellofemoral  Medial compartment, NOS  Lateral compartment, NOS  Cartilage injury, NOS | 43 (19.5%)  26 (11.8%)  11 (5.0%)  19 (8.6%)  20 (9.1%)  31 (14.0%)  14 (6.3%)  7 (3.2%)  4 (1.8%)  2 (0.9%) | 16 (14.8%)  4 (3.7%)  5 (4.6%)  4 (3.7%)  5 (4.6%)  23 (21.3%)  7 (6.5%)  9 (8.3%)  8 (7.4%)  1 (0.9%) | 59 (17.9%)  30 (9.1%)  16 (4.9%)  23 (7.0%)  25 (7.6%)  54 (16.4%)  21 (6.4%)  16 (4.9%)  12 (3.7%)  3 (0.9%) | Eliminated due to:   - high variability in how information documented and low preliminary performance metrics - lower priority for final use-case as able to obtain some information from ICD/CPT codes | X  X  X  X  X  X  X  X  X  X |  |
| *Articular cartilage procedures* | 22 (10.0%) | 8 (7.4%) | 30 (9.1%) |  |  |  |
| ORIF  Debridement/chondroplasty  Microfracture  OATS  MACI  Other | 0 (0%)  20 (9.1%)  1 (0.5%)  0 (0%)  0 (0%)  1 (0.5%) | 0 (0%)  8 (7.4%)  0 (0%)  0 (0%)  0 (0%)  0 (0%) | 0 (0%)  28 (8.5%)  1 (0.3%)  0 (0%)  0 (0%)  1 (0.3%) |  | X  X  X  X  X  X |  |
| PCL injury | 3 (1.4%) | 1 (0.9%) | 4 (1.2%) |  | X |  |
| *PCL procedure* | 2 (0.9%) | 1 (0.9%) | 2 (0.9%) |  |  |  |
| Reconstruction | 2 (0.9%) | 0 (0%) | 2 (0.6%) |  | X |  |
| Repair | 0 (0%) | 1 (0.9%) | 1 (0.3%) |  | X |  |
| MCL injury | 19 (8.6%) | 10 (9.3%) | 29 (8.8%) |  | X |  |
| *MCL procedure* | 3 (1.4%) | 5 (4.6%) | 8 (2.4%) |  |  |  |
| Reconstruction | 1 (0.5%) | 1 (0.9%) | 2 (0.6%) |  | X |  |
| Repair | 2 (0.9%) | 4 (3.7%) | 6 (1.8%) |  | X |  |
| LCL injury | 4 (1.8%) | 0 (0%) | 4 (1.2%) |  | X |  |
| *LCL procedure* | 0 (0%) | 0 (0%) | 0 (0%) |  |  |  |
| Reconstruction | 0 (0%) | 0 (0%) | 0 (0%) |  | X |  |
| Repair | 0 (0%) | 0 (0%) | 0 (0%) |  | X |  |
| PLC injury | 3 (1.4%) | 0 (0%) | 3 (0.9%) |  | X |  |
| PLC procedure | 2 (0.9%) | 0 (0%) | 2 (0.6%) |  | X |  |
| Synovectomy | 13 (5.9%) | 4 (3.7%) | 17 (5.2%) |  |  |  |
| ^1^ Auto/allograft included in respective counts  ^2^ Included for completeness but not an entity; no performance metrics calculated  ACL, anterior cruciate ligament; ARIF, arthroscopic reduction and internal fixation; BTB, bone patellar tendon bone; LCL, lateral collateral ligament; MACI, matrix-induced autologous chondrocyte implantation; MCL, medial collateral ligament; NOS, not otherwise specified; ORIF, open reduction and internal fixation; OATS, osteochondral autograft transfer system; PCL, posterior cruciate ligament; PLC, posterolateral corner | | | | | | |

Appendix 3 Initial Validation Set Performance Metrics

|  | **Initial Validation Set**  (n = 108) | | | | | | | |
| --- | --- | --- | --- | --- | --- | --- | --- | --- |
|  | **Exact Match** | | | | **Note Level Match** | | | |
|  | **n** | **Recall** | **Precision** | **F1** | **n** | **Recall** | **Precision** | **F1** |
| Date of birth | 108 | 1 | 1 | 1 | 108 | 0.991 | 0.991 | 0.991 |
| Date of surgery | 107 | 0.991 | 1 | 0.995 | 107 | 0.944 | 1 | 0.971 |
| Hospital | 24 | 0.792 | 0.864 | 0.826 | Dropped | | | |
| *Side of surgery* |  |  |  |  | 108 | 0.954 | 0.981 | 0.967 |
| Right knee | 268 | 0.679 | 0.905 | 0.776 | 58 | 0.966 | 0.949 | 0.957 |
| Left knee | 215 | 0.637 | 0.797 | 0.708 | 50 | 0.980 | 0.925 | 0.951 |
| *General anesthesia* |  |  |  |  | Dropped | | | |
| Endotracheal | 12 | 0.25 | 0.429 | 0.316 | Dropped | | | |
| Laryngeal mask | 13 | 0.692 | 1 | 0.818 |  |  |  |  |
| General, NOS | 171 | 0.959 | 0.976 | 0.968 |  |  |  |  |
| *Regional anesthesia* |  |  |  |  | Dropped | | | |
| Spinal | 3 | 0 | NA | 0 | Dropped | | | |
| Epidural | 0 | - | - | - |  |  |  |  |
| Femoral block | 29 | 0.793 | 0.958 | 0.868 |  |  |  |  |
| Sciatic block | 0 | - | - | - |  |  |  |  |
| Adductor canal block | 0 | - | - | - |  |  |  |  |
| *Adductor/sciatic block* |  |  |  |  |  |  |  |  |
| Femoral/sciatic block | 2 | 0.5 | 1 | 0.667 |  |  |  |  |
| *Femoral/spinal* |  |  |  |  |  |  |  |  |
| Regional, NOS | 30 | 0.367 | 0.55 | 0.44 |  |  |  |  |
| Local anesthesia | 2 | 0 | NA | 0 | Dropped | | | |
| *Tourniquet use* |  |  |  |  | 81 | 0.840 | 0.971 | 0.901 |
| Tourniquet used | 24 | 0.292 | 0.259 | 0.275 | 67 | 0.910 | 0.968 | 0.938 |
| Tourniquet not used | 13 | 0.385 | 0.714 | 0.5 | 14 | 0.500 | 1 | 0.667 |
| Presence of tourniquet time | 40 | 0.875 | 0.946 | 0.909 | Combined with tourniquet use | | | |
| Presence of tourniquet pressure | 58 | 0.862 | 0.926 | 0.893 |  |  |  |  |
| *ACL diagnosis* |  |  |  |  | 107 | 0.972 | 0.963 | 0.967 |
| Tear | 346 | 0.506 | 0.543 | 0.524 | 97 | 0.990 | 0.970 | 0.980 |
| Recurrent tear | 27 | 0.333 | 1 | 0.5 | 10 | 0.400 | 1 | 0.571 |
| Avulsion fracture | 0 | - | - | - | 0 | - | - | - |
| *ACL procedure^1^*  *ACL procedure – recurrent/revision combo^2^* |  |  |  |  | 107  107 | 0.935  0.972 | 0.962  1 | 0.948  0.986 |
| Reconstruction | 179 | 0.894 | 0.856 | 0.874 | 97 | 0.969 | 0.922 | 0.945 |
| *Reconstruction – combo^2^* |  |  |  |  | 97 | 0.969 | 1 | 0.984 |
| Repair | 0 | - | - | - | 0 | - | - | - |
| Debridement of ACL | 0 | - | - | - | 0 | - | - | - |
| ARIF | 0 | - | - | - | 0 | - | - | - |
| Augmentation | 0 | - | - | - | 0 | - | - | - |
| Revision ACLR | 19 | 0.421 | 0.615 | 0.5 | 7 | 0.857 | 0.667 | 0.750 |
| *Recurrent/ revision combo^2^* |  |  |  |  | 10 | 1 | 1 | 1 |
| *Graft debridement and tunnel graft* | Added for final test set performance metric calculations | | | | | | | |
| Graft debridement | 3 | 0 | 0 | 0 | 3 | 0 | 0 | 0 |
| ACL tunnel grafting | Added for final test set performance metric calculations | | | | | | | |
| ACL prior contralateral injury | 0 | - | - | - | Dropped | | | |
| Notchplasty | 44 | 0.727 | 0.78 | 0.753 | 37 | 0.946 | 1 | 0.972 |
| *ACL graft type – allograft/autograft^3^* |  |  |  |  | 105 | 0.838 | 0.946 | 0.889 |
| *ACL graft type – specific type^4^* |  |  |  |  | 105 | 0.790 | 0.892 | 0.838 |
| *ACL autograft* |  |  |  |  | 69 | 0.884 | 0.968 | 0.924 |
| BTB autograft | 62 | 0.677 | 0.894 | 0.771 | 32 | 0.906 | 0.935 | 0.921 |
| Hamstring tendon autograft | 52 | 0.538 | 0.7 | 0.609 | 22 | 0.818 | 1 | 0.900 |
| Quadriceps tendon autograft | 34 | 0.882 | 0.968 | 0.923 | 15 | 0.933 | 1 | 0.966 |
| *ACL allograft* |  |  |  |  | 38 | 0.658 | 0.758 | 0.704 |
| Achilles tendon allograft | 7 | 0.857 | 1 | 0.923 | 5 | 1 | 1 | 1 |
| BTB allograft | 9 | 0.444 | 0.8 | 0.571 | 8 | 0.500 | 1 | 0.667 |
| Hamstring tendon allograft | 11 | 0.545 | 1 | 0.706 | 6 | 0.833 | 1 | 0.909 |
| Peroneus longus tendon allograft | 2 | 0 | NA | 0 | 2 | 0 | NA | 0 |
| Anterior tibial tendon allograft | 5 | 0.2 | 0.5 | 0.286 | 4 | 0 | NA | 0 |
| Posterior tibial tendon allograft | 7 | 0.714 | 0.833 | 0.769 | 4 | 0.750 | 0.750 | 0.750 |
| Allograft, NOS | 32 | 0.469 | 0.75 | 0.577 | 9 | 0.778 | 0.538 | 0.636 |
| *ACL hybrid graft^5^* |  |  |  |  | 2 | 0.500 | 0.333 | 0.400 |
| ACL graft width | 106 | 0.434 | 0.613 | 0.508 | 76 | 0.487 | 0.712 | 0.578 |
| Femoral tunnel diameter | 99 | 0.455 | 0.523 | 0.486 | Dropped | | | |
| Tibial tunnel diameter | 99 | 0.465 | 0.505 | 0.484 | Dropped | | | |
| *Meniscal tear^6^* |  |  |  |  | 71 | 0.817 | 0.853 | 0.835 |
| Medial meniscus tear | 149 | 0.631 | 0.797 | 0.704 | 49 | 0.918 | 0.978 | 0.947 |
| Lateral meniscus tear | 130 | 0.669 | 0.75 | 0.707 | 47 | 0.872 | 0.872 | 0.872 |
| *Medial meniscus procedure* |  |  |  |  | 48 | 0.771 | 1 | 0.871 |
| Meniscectomy | 41 | 0.585 | 0.774 | 0.667 | 30 | 0.833 | 1 | 0.909 |
| Repair | 32 | 0.625 | 0.952 | 0.755 | 19 | 0.684 | 1 | 0.813 |
| Transplant | 0 | - | - | - | 0 | - | - | - |
| *Lateral meniscus procedure* |  |  |  |  | 41 | 0.829 | 1 | 0.907 |
| Meniscectomy | 43 | 0.535 | 0.742 | 0.622 | 29 | 0.793 | 1 | 0.885 |
| Repair | 25 | 0.76 | 0.905 | 0.826 | 12 | 0.917 | 0.917 | 0.917 |
| Transplant | 0 | - | - | - | 0 | - | - | - |
| *Articular cartilage injury* |  |  |  |  | Dropped | | | |
| Medial femoral condyle | 22 | 0.273 | 0.545 | 0.364 | Dropped | | | |
| Lateral femoral condyle | 4 | 0.5 | 1 | 0.667 |  |  |  |  |
| Trochlea | 5 | 0 | NA | 0 |  |  |  |  |
| Medial tibial plateau | 4 | 0 | 0 | 0 |  |  |  |  |
| Lateral tibial plateau | 7 | 0 | NA | 0 |  |  |  |  |
| Patella | 29 | 0.138 | 0.571 | 0.222 |  |  |  |  |
| Patellofemoral | 8 | 0 | NA | 0 |  |  |  |  |
| Medial compartment, NOS | 9 | 0 | NA | 0 |  |  |  |  |
| Lateral compartment, NOS | 8 | 0 | NA | 0 |  |  |  |  |
| Cartilage injury, NOS | 2 | 0 | NA | 0 |  |  |  |  |
| *Articular cartilage procedures* |  |  |  |  | Dropped | | | |
| ORIF | 0 | - | - | - | Dropped | | | |
| Debridement/chondroplasty | 14 | 0.286 | 0.667 | 0.4 |  |  |  |  |
| Microfracture | 0 | - | - | - |  |  |  |  |
| OATS | 0 | - | - | - |  |  |  |  |
| MACI | 0 | - | - | - |  |  |  |  |
| Other | 0 | - | - | - |  |  |  |  |
| PCL injury | 5 | 0 | NA | 0 | Dropped | | | |
| *PCL procedure* |  |  |  |  | Dropped | | | |
| Reconstruction | 0 | - | - | - | Dropped | | | |
| Repair | 2 | 0 | NA | 0 |  |  |  |  |
| MCL injury | 22 | 0.364 | 0.571 | 0.444 | Dropped | | | |
| *MCL procedure* |  |  |  |  | Dropped | | | |
| Reconstruction | 2 | 0 | NA | 0 | Dropped | | | |
| Repair | 7 | 0 | NA | 0 |  |  |  |  |
| LCL injury | 0 | - | - | - | Dropped | | | |
| *LCL procedure* |  |  |  |  | Dropped | | | |
| Reconstruction | 0 | - | - | - | Dropped | | | |
| Repair | 0 | - | - | - |  |  |  |  |
| PLC injury | 0 | - | - | - | Dropped | | | |
| PLC procedure | 0 | - | - | - | Dropped | | | |
| Synovectomy | 7 | 0.571 | 0.667 | 0.615 | 4 | 0.750 | 1.000 | 0.857 |
| Italicized rows are not individual entities, rather combinations or collapsing of individual entities listed in adjacent section at the note level  ^1^ACL procedure – categories include ACL reconstruction, Debridement of ACL, Augmentation, ACL graft debridement and/or tunnel graft, and ACL revision; ACL repair and ARIF not present in test set  ^2^ACL procedure – recurrent/revision combo: Same as overall ACL procedure entity but instead of a revision category, created a combined category for individuals with indication of recurrent tears, revision, debridement of grafts or bone tunnel grafting as these individuals will be excluded from final use case  ^3^ ACL graft type – allograft/ autograft: collapsed specific graft types to categorize each note as allograft, autograft, or hybrid (3 categories)  ^4^ ACL graft type – specific type: categorized each note by specific autograft and/or allograft type (11 categories)  ^5^ ACL hybrid graft: Autograft and allograft types included in respective counts  ^6^ Meniscal tear – specific location: Three categories including medial meniscus tear, lateral meniscus tear, both medial and lateral meniscal tears  ACL, anterior cruciate ligament; ARIF, arthroscopic reduction and internal fixation; BTB, bone patellar tendon bone; LCL, lateral collateral ligament; MACI, matrix-induced autologous chondrocyte implantation; MCL, medial collateral ligament; NOS, not otherwise specified; ORIF, open reduction and internal fixation; OATS, osteochondral autograft transfer system; PCL, posterior cruciate ligament; PLC, posterolateral corner | | | | | | | | |
